# Activation of platelet-rich plasma by pulse electric fields: Voltage, pulse width and calcium concentration can be used to control and tune the release of growth factors, serotonin and hemoglobin

**DOI:** 10.1371/journal.pone.0249209

**Published:** 2021-04-23

**Authors:** Bogdan Neculaes, Andrew L. Frelinger, Anja J. Gerrits, Thomas Gremmel, Emma E. Forde, Steven Klopman, Sabrina L. Carmichael, Alan D. Michelson

**Affiliations:** 1 GE Research, Niskayuna, NY, United States of America; 2 Center for Platelet Research Studies, Dana-Farber/Boston Children’s Cancer and Blood Disorders Center, Harvard Medical School, Boston, MA, United States of America; 3 Department of Internal Medicine II, Medical University of Vienna, Vienna, Austria; 4 Department of Internal Medicine I, Cardiology and Intensive Care Medicine, Landesklinikum Mistelbach-Gaenserndorf, Mistelbach, Austria; Institut d’Investigacions Biomediques de Barcelona, SPAIN

## Abstract

Activated platelet-rich plasma (PRP) has been used in the clinical settings of wound healing and regenerative medicine, with activation typically induced by the addition of bovine thrombin. To eliminate issues with availability, cost and potential side effects associated with bovine thrombin, *ex vivo* PRP activation using pulse electric fields (PEF) has been proposed and demonstrated. The present study characterizes the effect of PEF voltage and pulse width, in combination with a range of calcium concentrations, on clot formation, growth factor release, and serotonin (5-HT) release from dense granules. The main findings are: 1) increasing calcium concentrations with most PEF conditions leads to increased levels of PDGF and 5-HT release; 2) whether EGF levels increase or decrease with increasing calcium concentration depends on the specific PEF parameters; 3) the pattern of PDGF and EGF levels in supernatants suggest that these molecules are localized differently within platelets; 4) significant levels of PDGF, EGF, and 5-HT can be released without inducing clot formation or hemoglobin release. In conclusion, voltage, pulse width and calcium concentration can be used to control and tune the release of growth factors, serotonin and hemoglobin from PEF-activated PRP. Because growth factor requirements vary for different types of wounds and for wounds at different stages of healing, the unique balance of factors in supernatants of PEF-activated PRP may provide more clinically advantageous than the current standard of bovine thrombin-activated PRP.

## Introduction

Platelet rich plasma has been explored for various clinical applications, leveraging the growth factors and proteins released by platelets upon activation [1–6], Promotion of wound healing by clinically administered platelet-rich plasma (PRP) includes several steps: blood draw from the patient; PRP separation from whole blood; activation–typically with bovine thrombin (although there is no clinical standard for bovine thrombin activation); topical application of the activated PRP on the wound. For specific PRP applications, the activation step is omitted, and non-activated PRP is directly injected at the site of the injury. These workflows attempt to harvest the effects on the wound healing cascade of growth factors released from platelets. For injections with non-activated PRP, it is considered that the platelets are activated in vivo, by the collagen at the site of the injury. The clinical applications of PRP in wound healing and regenerative medicine include diabetic foot ulcers [3], cardiac surgery [4], orthopedics and sports medicine [7], dermatology and hair loss [8–10].

*In vitro* activation of PRP using pulse electric fields (PEF) offers an alternative to bovine thrombin activation: an instrument-based, easy-to-standardize method, without the challenges of bovine thrombin (side effects, cost, workflow); it should be noted that autologous thrombin is another alternative to bovine thrombin, although this additional biomaterial needs to be generated at the bed side; the activation potential of autologous thrombin is an area of ongoing research. Initial pre-clinical studies produced promising wound healing results using PRP activated via PEF [11]. Mechanistically, it is believed that PEF may cause platelet activation with growth factor release and clotting via Ca transport and platelet membrane and intracellular organelle electro-permeabilization [12]. However, subsequent research discovered that PEF treatment of PRP enables growth factor release with or without clotting [13]–a unique feature that adds additional clinical functionality compared to the use of bovine thrombin. One could envision PEF-induced growth factor release and clotting of PRP for use in topical applications, and PEF-induced growth factor release without clotting of PRP for use in injections to accelerate the healing of injured tendons, ligaments, muscles and joints.

The work presented here studies clotting features and platelet alpha granule content (growth factors) and platelet dense granule content (serotonin [5-hydroxytryptamine (5-HT)]) release at multiple electric pulse (five types of electrical pulses) and CaCl_2_ parameters (four CaCl_2_ conditions). In addition, because PRP prepared by a number of commercial systems contains significant numbers of red blood cells (0.2–3.2 million RBCs per μL PRP) [14] we investigate the effect of PEF on RBC lysis and release of hemoglobin which can catalyze oxidation of neighboring molecules, generate free radicals, and lead to cell death [15–17].

## Methods

### Donors, blood collection and preparation of PRP

This study was reviewed and approved by the Boston Children’s Hospital Committee on Clinical Investigation and all subjects provided written informed consent. Healthy volunteers (n = 3) were qualified for enrollment if they were aged ≥18 years, free of aspirin or other antiplatelet medication (≥10 days), and free of all other non-steroidal anti-inflammatory drugs (≥ 3 days). Following a 2 mL discard, 120 mL of blood was collected from each of 3 volunteers into 1/10^th^ volume of acid-citrate-dextrose solution A (ACD-A). PRP was prepared according to the manufacturer’s recommendation using the Harvest SmartPreP2 System (Harvest Technologies, Plymouth, MA, USA) with two 60 mL cartridges. The resultant PRP was pooled prior to further treatment. Complete blood cell counts were performed on the ACD-anticoagulated whole blood and the concentrated PRP in a Sysmex XN Hematology Analyzer. Prior to activation, to increase the total number of conditions that could be evaluated for each donor, the PRP was diluted with platelet-poor plasma to obtain a total volume of 24 mL.

### Study design

PRP activation by PEF (conditions described below) was evaluated in the presence of buffer (no added CaCl_2_) or added CaCl_2_ (5.35 mM, 11.61 mM, or 17.04 mM) calculated to result in final free ionized calcium levels of 0.05 mM, 0.2 mM, 0.8 mM, or 3.0 mM. Controls included unactivated PRP, PRP activated with bovine thrombin (1 U/mL final concentration, Biopharm Laboratories LLC, Bluffdale, UT, USA) in the presence of 17 mM added CaCl_2_, and PRP lysed by freezing and thawing three times. Endpoints measured included: 1) clot formation kinetics and strength by thromboelastography (TEG); 2) hemolysis as reflected by hemoglobin release; 3) release of epidermal growth factor (EGF) *vs*. a representative known alpha granule constituent, platelet-derived growth factor (PDGF); 4) serotonin (5-HT) release into the supernatant from platelet dense granules. All endpoints except TEG were measured in samples taken 15 min after activation.

For analysis by TEG, 360 μL of activated PRP was quickly transferred to the TEG cup and recordings initiated immediately. A separate independent sample was activated under identical conditions and allowed to stand 15 min at room temperature following activation, then clots were removed using the wooden handle of a cotton swab and the resulting serum was frozen at -80°C for later evaluation of released hemoglobin, growth factors, and 5-HT.

### Pulse electric field stimulation of PRP

**Fig 1:**
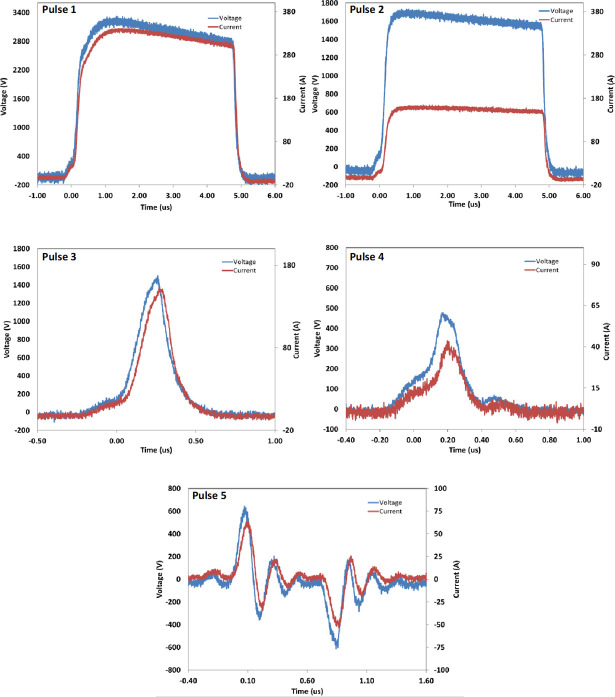
Pulse Electric Field (PEF) conditions used in this study.

Electrical stimulation of PRP was performed using a specially designed instrument prototype (GE Research, Niskayuna, NY, USA), which has previously been described [18]. The instrument takes into account the specific electrical properties of PRP which is typically more conductive than the buffers used in electroporation. Concentrated PRP (~500 μL) was placed in a 2 mm electroporation cuvette (Molecular BioProducts, San Diego, CA, USA), pre-loaded with 1/100^th^ volume buffer or CaCl_2_ (5.35 mM, 11.61 mM, and 17.04 mM final concentration), and exposed to one of five PEF conditions (Pulses 1–5, see **Fig 1**): Pulse 1: 3.3 kV peak voltage, pulse duration 5 us, one pulse applied; Pulse 2: 1.6 kV peak voltage, pulse duration 5 us, one pulse applied; Pulse 3: 1.5 kV peak voltage, pulse duration 450 ns, one pulse applied; Pulse 4: 500 V peak voltage, pulse duration 350 ns, one pulse applied; Pulse 5: 80 pairs of bipolar pulses, one pair per second, ~ 150 ns pulse duration and ~ 650 V peak voltage for each bipolar pulses, the two bipolar pulses separated by ~ 600 ns.

A Tektronix DPO4104 oscilloscope and a Tektronix P6015A high voltage probe were used to measure the voltage pulses applied to cuvettes with PRP for activation.

### Thromboelastography

Immediately following exposure of PRP to activating conditions, 360 μL of treated PRP was placed in a TEG cup and analyzed by a TEG 5000 Hemostasis Analyzer System (Haemonetics Corporation, Braintree, MA, USA). Clotting kinetics and characteristics were followed for 30 minutes.

### Spectrophotometric determination of hemoglobin

Plasma hemoglobin was measured using a spectrophotometric method [19]. Briefly, normal donor blood was centrifuged for 10 min at 800 x g and the PRP upper layer removed, leaving packed RBCs. Total hemoglobin in the packed RBCs was measured using a Sysmex XN automated cell analyzer. Standards with different know hemoglobin concentrations were then generated from this sample by dilution in 10 mM HEPES, 0.15 M sodium chloride, supplemented with 7% bovine serum albumin. Standards and unknowns were mixed with 0.04% ammonium hydroxide and allowed to stand at room temperature for 1 hour before reading the absorbance at 576 nm on a Molecular Dynamics 96 well plate reader. An initial subjective estimate of the hemoglobin in samples was made by comparing the color of the sample to that of the standards. Based on this comparison, for samples appearing dark red in color, 5 μL of sample was mixed with 45 μL 0.04% ammonium hydroxide and compared to 5 μL of standards mixed with 45 μL 0.04% ammonium hydroxide. For samples appearing to have less hemolysis, 20 μL of sample was mixed with 180 μL 0.04% ammonium hydroxide and compared to standards prepared in the same way. All samples were within the linear range of the standard curve after dilution.

### Measurement of EGF, PDGF, and 5-HT

Levels of EGF, PDGF and 5-HT in the supernatants of the treated PRP were measured using commercially available ELISA kits (EGF and PDGF R&D Systems, Minneapolis, MN, USA; 5-HT, BA E-5900, Rocky Mountain Diagnostics, Colorado Springs, CO, USA). N = 3 for each data point.

## Results

Blood cell counts for the collected whole blood and prepared PRP are shown in **Table 1**.

**Table 1:** Cell composition and fold concentration of PRP prepared using the Harvest system.

Parameter	Whole Blood	PRP	Diluted PRP	Fold-Concentration of PRP Compared to Whole Blood
WBC (×10^9^/L)	4.83 ± 0.85	12.63 ± 1.42	10.22 ± 1.05	2.65 ± 0.4
RBC (×10^12^/L	4.23 ± 0.51	1.68 ± 0.36	1.38 ± 0.2	0.41 ± 0.13
Hgb (g/dL)	12.7 ± 1.92	4.93 ± 1	4.03 ± 0.64	0.4 ± 0.14
HCT (%)	37.7 ± 4.2	15.5 ± 3.7	12.7 ± 2.3	0.42 ± 0.14
PLT (×10^9^/L)	228 ± 73	944 ± 298	777 ± 210	4.14 ± 0.11

Abbreviations: HCT, hematocrit; PLT, platelet; PPP, platelet-poor plasma; PRP, platelet-rich plasma; RBC, red blood cell; WBC, white blood cell. Data are mean ± SD, n = 3.

**Fig 2:**
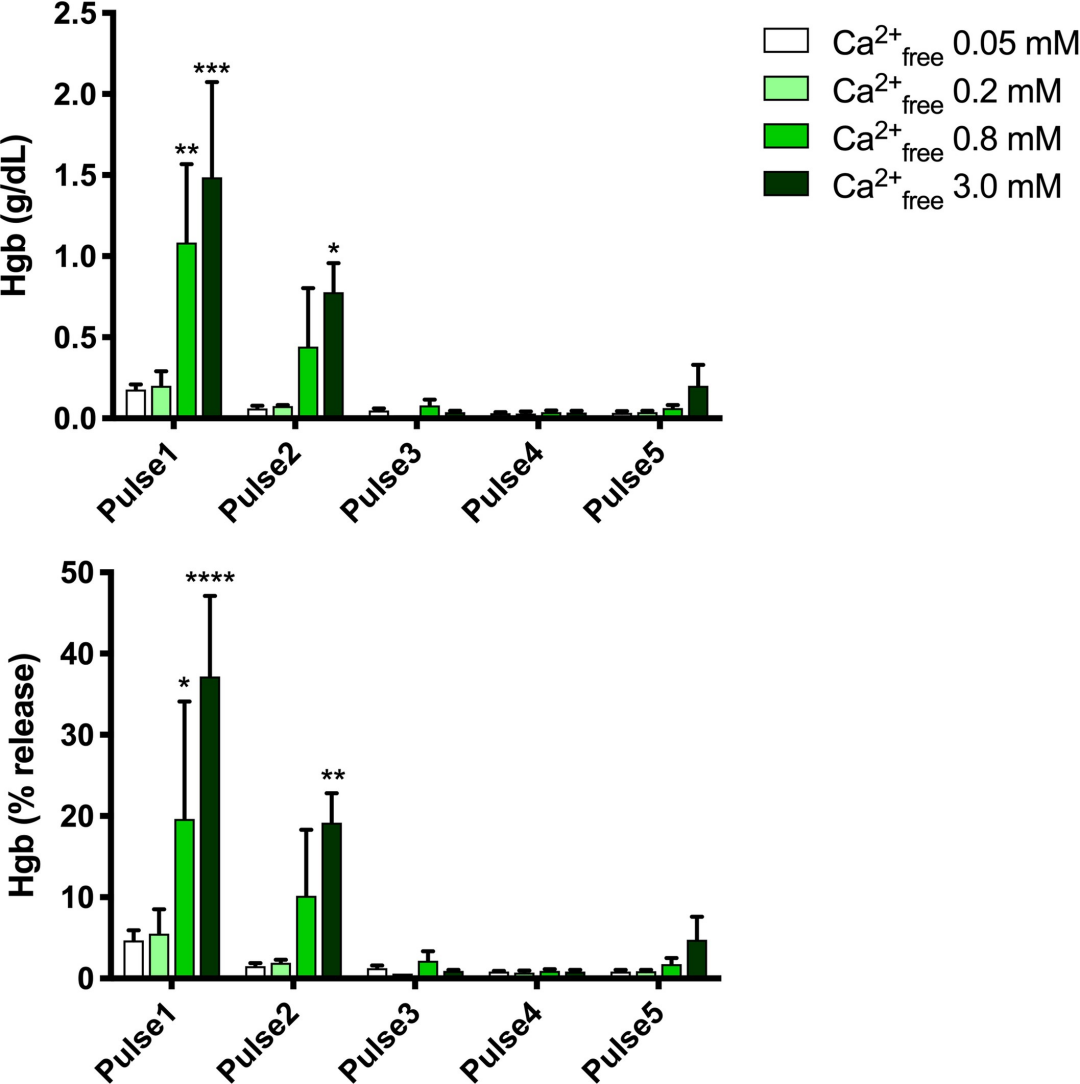
Hemoglobin in supernatants of electrical activated samples. Upper panel, g/dL, lower panel, % of total hemoglobin. Results are means ± SEM, n = 3. Asterisks indicate significant differences within each pulse treatment for 0.2, 0.8 and 3.0 mM Ca^2+^_free_ vs. 0.05 Ca^2+^_free_. *p<0.05, **<0.01, ***<0.001, **** <0.0001, Tukey’s multiple comparisons test adjusted for multiplicity.

Platelet count was increased ~4-fold and WBC count was increased ~2.6-fold in PRP compared to levels in whole blood, while RBC count and hematocrit in PRP were ~40% of those in whole blood. The amounts of hemoglobin present in the supernatants of activated samples are shown in **Table 2** and **Fig 2**.

**Table 2:** Hemoglobin in supernatants of activated samples.

**A**				
	**Ca**^**2+**^_**free**_ **0.05 mM**	**Ca**^**2+**^_**free**_ **0.2 mM**	**Ca**^**2+**^_**free**_ **0.8 mM**	**Ca**^**2+**^_**free**_ **3.0 mM**
**Pulse 1**	0.18 ± 0.05	0.2 ± 0.16	0.68 ± 0.63	1.49 ± 0.83
**Pulse 2**	0.06 ± 0.03	0.08 ± 0.01	0.44 ± 0.62	0.78 ± 0.31
**Pulse 3**	0.05 ± 0.02	0.02	0.08 ± 0.06	0.04 ± 0.01
**Pulse 4**	0.03 ± 0.01	0.03 ± 0.02	0.04 ± 0.02	0.04 ± 0.02
**Pulse 5**	0.03 ± 0.02	0.04 ± 0.02	0.06 ± 0.03	0.2 ± 0.22
**B**				
	**Ca**^**2+**^_**free**_ **0.05 mM**	**Ca**^**2+**^_**free**_ **0.2 mM**	**Ca**^**2+**^_**free**_ **0.8 mM**	**Ca**^**2+**^_**free**_ **3.0 mM**
**Pulse 1**	4.7 ± 2.2	5.5 ± 5.3	19.7 ± 20.4	37.2 ± 14
**Pulse 2**	1.51 ± 0.63	1.9 ± 0.6	10.2 ± 14	19.2 ± 6.3
**Pulse 3**	1.26 ± 0.6	0.6	2.2 ± 2.1	0.9 ± 0.2
**Pulse 4**	0.8 ± 0.16	0.8 ± 0.4	0.9 ± 0.4	0.9 ± 0.3
**Pulse 5**	0.82 ± 0.39	0.9 ± 0.3	1.8 ± 1	4.8 ± 4.9

Column headings show the calculated free calcium (Ca^2+^_free_) present in each sample. A: g/dL, B: % of total. Results shown are means ± SD, n = 3.

Release of hemoglobin was dependent on both the PEF condition and the final free calcium concentration, with greater amounts of hemoglobin release at higher voltage settings and higher calcium concentrations. Lower time settings (Pulse 3, Pulse 4 *vs*. Pulse 2) resulted in virtually no release of hemoglobin from RBCs even at high calcium concentrations.

**Fig 3:**
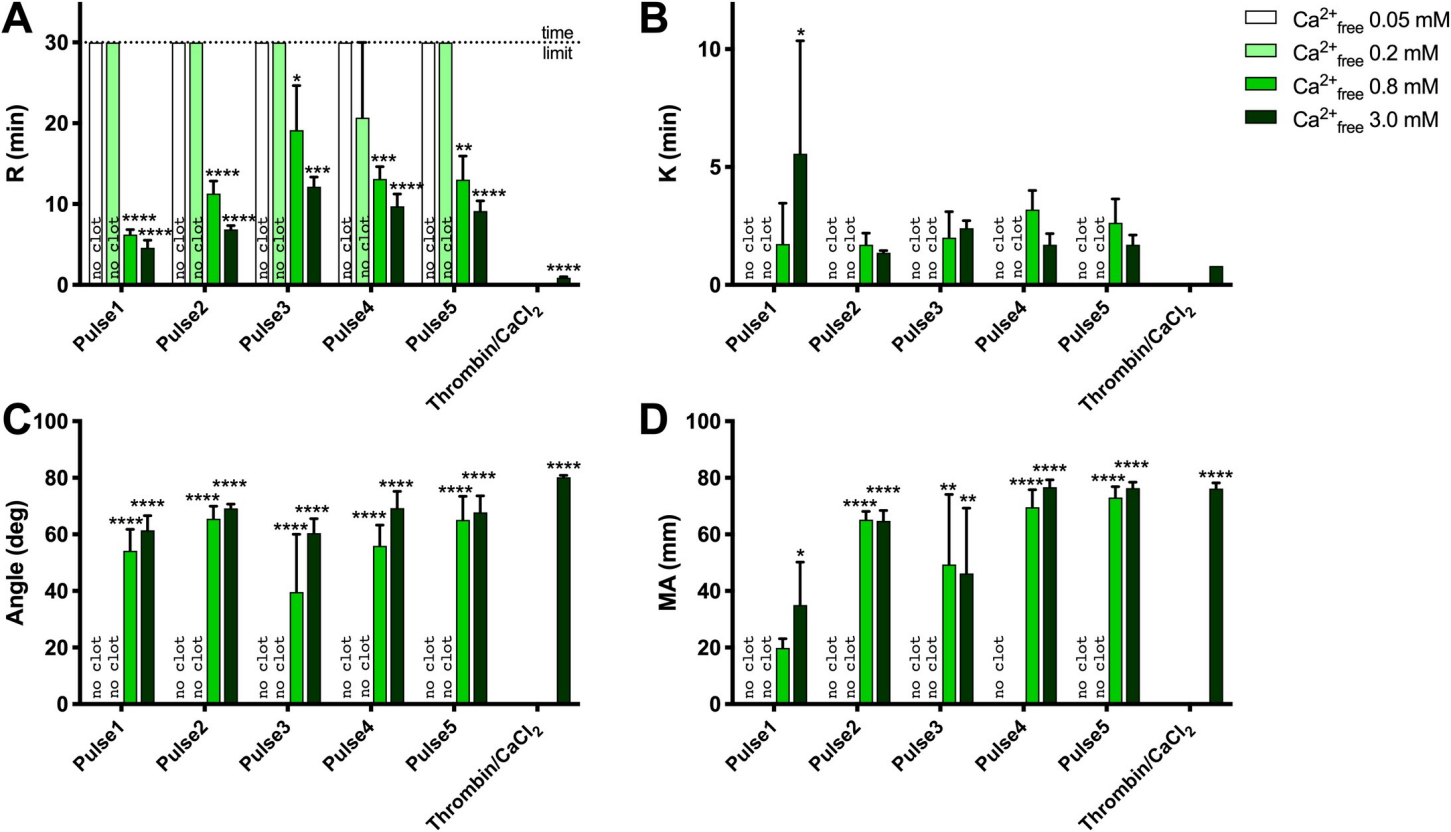
TEG analysis of clot formation and strength. A) R (min), reaction time for first significant clot formation, B) K (min), time required to achieve a pre-specified clot strength, C) Angle (deg), rate of clot development, D) maximum amplitude (MA, mm), maximum clot strength (related to elastic modulus). Results are means ± SEM, n = 3. Asterisks indicate significant differences within each pulse treatment for 0.2, 0.8 and 3.0 mM Ca^2+^_free_ vs. 0.05 Ca^2+^_free_. *p<0.05, **<0.01, ***<0.001, **** <0.0001, Tukey’s multiple comparisons test adjusted for multiplicity.

Clot formation as measured by thromboelastography is shown in **Fig 3** and **Table 3**.

**Table 3:** Clot formation and strength parameters.

**A. R (min)**				
	Ca^2+^_free_ 0.05 mM	Ca^2+^_free_ 0.2 mM	Ca^2+^_free_ 0.8 mM	Ca^2+^_free_ 3.0 mM
**Pulse 1**	30 ± 0	30 ± 0	6.23 ± 1.03	4.6 ± 1.59
**Pulse 2**	30 ± 0	30 ± 0	11.3 ± 2.7	6.87 ± 0.8
**Pulse 3**	30 ± 0	30 ± 0	19.13 ± 9.56	12.13 ± 2.11
**Pulse 4**	30 ± 0	20.7 ± 16.11	13.1 ± 2.65	9.73 ± 2.61
**Pulse 5**	30 ± 0	30 ± 0	13.03 ± 5.06	9.13 ± 2.21
**B. K (min)**				
	Ca^2+^_free_ 0.05 mM	Ca^2+^_free_ 0.2 mM	Ca^2+^_free_ 0.8 mM	Ca^2+^_free_ 3.0 mM
**Pulse 1**	0 ± 0	0 ± 0	1.73 ± 3	5.57 ± 8.29
**Pulse 2**	0 ± 0	0 ± 0	1.7 ± 0.85	1.37 ± 0.15
**Pulse 3**	0 ± 0	0 ± 0	2 ± 1.91	2.4 ± 0.56
**Pulse 4**	0 ± 0	0 ± 0	3.2 ± 1.4	1.7 ± 0.82
**Pulse 5**	0 ± 0	0 ± 0	2.63 ± 1.76	1.7 ± 0.72
**C. Angle (deg)**				
	Ca2^+^_free_ 0.05 mM	Ca^2+^_free_ 0.2 mM	Ca^2+^_free_ 0.8 mM	Ca^2+^_free_ 3.0 mM
**Pulse 1**	0 ± 0	0 ± 0	54.2 ± 13.2	61.47 ± 8.95
**Pulse 2**	0 ± 0	0 ± 0	65.5 ± 7.73	69.2 ± 2.65
**Pulse 3**	0 ± 0	0 ± 0	39.6 ± 35.43	60.43 ± 8.92
**Pulse 4**	0 ± 0	0 ± 0	55.97 ± 12.69	69.3 ± 10.23
**Pulse 5**	0 ± 0	0 ± 0	65.13 ± 14.39	67.77 ± 10.13
**D. MA (mm)**				
	Ca^2+^_free_ 0.05 mM	Ca^2+^_free_ 0.2 mM	Ca^2+^_free_ 0.8 mM	Ca^2+^_free_ 3.0 mM
**Pulse 1**	0 ± 0	0 ± 0	19.9 ± 5.6	35.03 ± 26.34
**Pulse 2**	0 ± 0	0 ± 0	65.17 ± 5.05	64.77 ± 6.44
**Pulse 3**	0 ± 0	0 ± 0	49.33 ± 42.88	72.6 ± 5.96
**Pulse 4**	0 ± 0	0 ± 0	68.3 ± 8.96	76.7 ± 4.52
**Pulse 5**	0 ± 0	0 ± 0	73.07 ± 6.58	76.37 ± 3.61

Results shown are means ± SD, n = 3.

Regardless of activating conditions, clots were not detected by thromboelastography with no added calcium (estimated free Ca++ [Ca^2+^_Free_] 0.03–0.06 mM) and with 5.35 mM added calcium (estimated Ca^2+^_Free_ 0.2 mM). Clot formation occurred more quickly with 17 mM than with 11.6 mM added calcium chloride (shorter R times). However maximal clot strength achieved was similar. Clot formation with bovine thrombin was more rapid than with PEF but, again, maximal clot strength (MA) was similar for PEF *vs*. thrombin (**Fig 3**).

### Release of EGF, PDGF, and 5-HT

**Fig 4:**
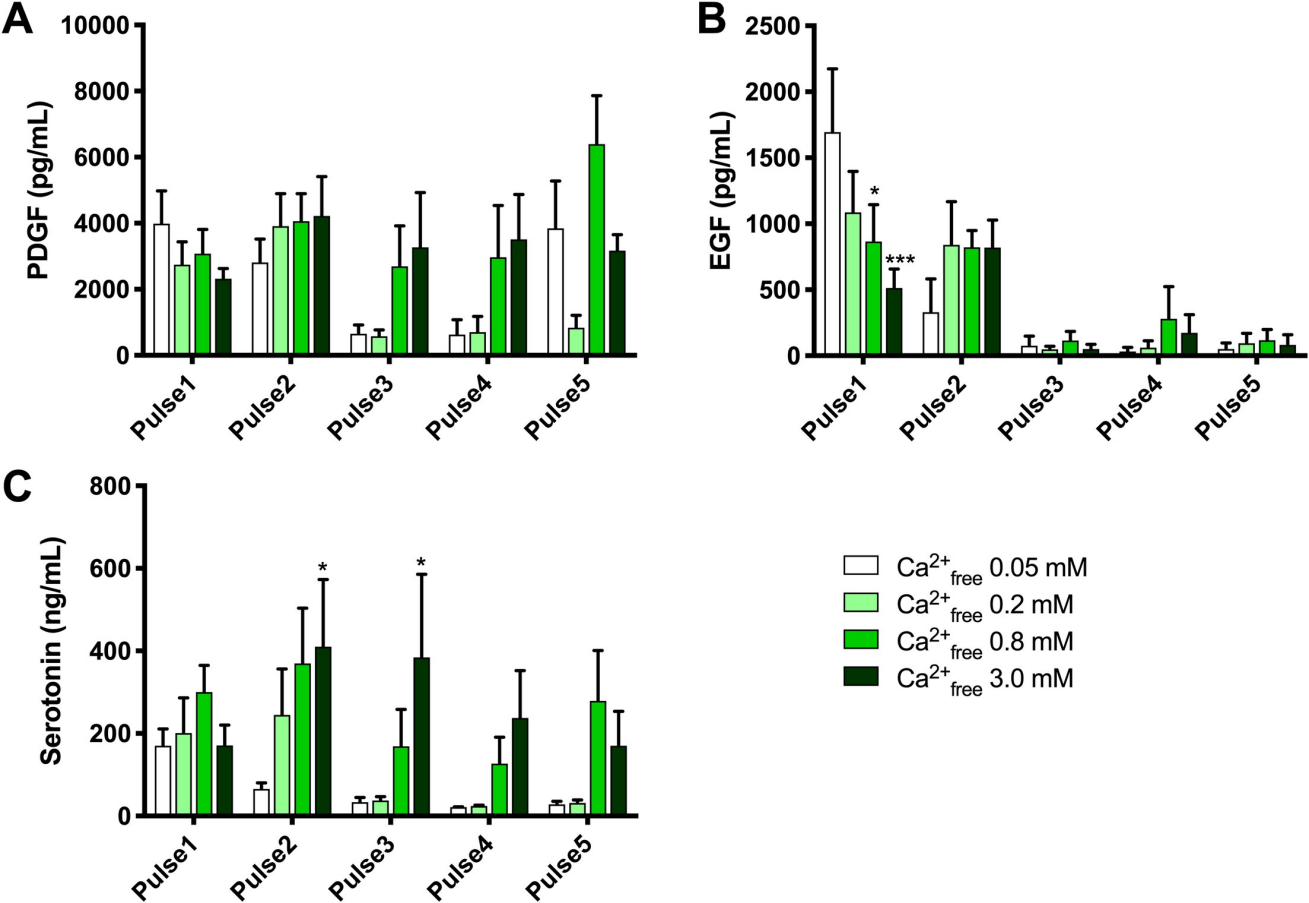
PDGF, EGF and 5-HT in supernatants of electrical activated samples. Results are means ± SEM, n = 3. Abbreviations: EGF, epidermal growth factor; PDGF, platelet-derived growth factor; PEF, pulse electric field. Asterisks indicate significant differences within each pulse treatment for 0.2, 0.8 and 3.0 mM Ca^2+^_free_ vs. 0.05 Ca^2+^_free_. *p<0.05, **<0.01, ***<0.001, **** <0.0001, Tukey’s multiple comparisons test adjusted for multiplicity.

**Fig 5:**
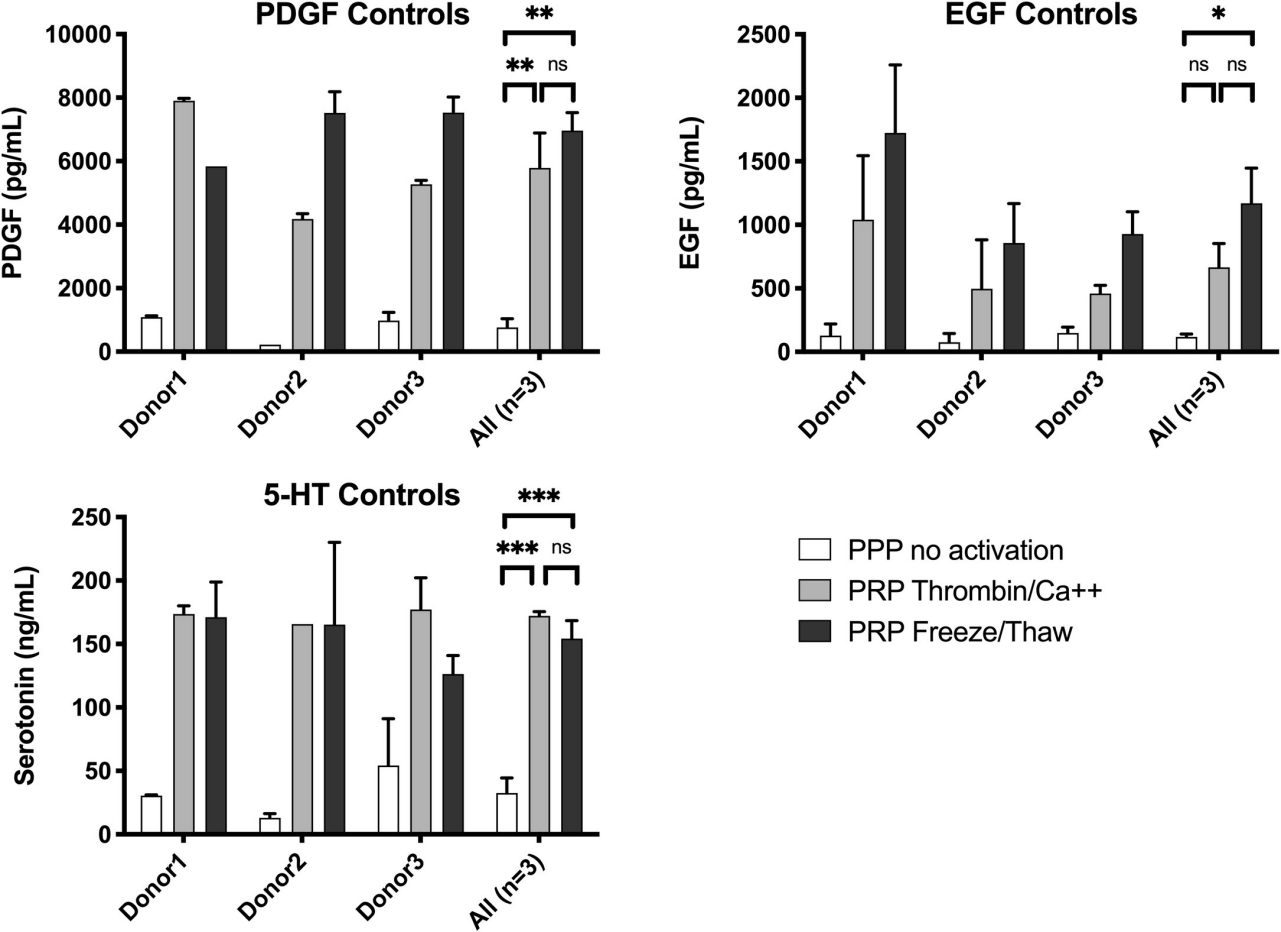
PDGF, EGF, and 5-HT in supernatants of control samples: Negative controls (no activation) and positive controls (thrombin activation and freeze / thaw). Results are means ± SEM, n = 3. Abbreviations: EGF, epidermal growth factor; Hgb, hemoglobin; PDGF, platelet-derived growth factor. Asterisks indicate significant differences in the amount of PDGF, EGF and 5-HT in the supernatants of un-activated, thrombin/Ca++ activated or freeze/thaw treated PRP. *p<0.05, **<0.01, ***<0.001 by ANOVA with Tukey’s multiple comparisons test adjusted for multiplicity.

PDGF is present in platelet alpha granules whereas the localization of EGF within platelets is less clear [20]. 5-HT is present in platelet dense granules [20]. The levels of each of these molecules in supernatants following PEF activation of PRP are shown in **Fig 4** and the levels released by bovine thrombin activation and freeze/thaw treatment are shown in **Fig 5**.

PDGF levels with PEF and high calcium (**Fig 4**) were ~50% of those released by thrombin or freeze/thaw treatment (**Fig 5**). PDGF decreased slightly with increasing calcium with Pulse 1 and increased slightly with increasing calcium with Pulse 2. In contrast, PEF Pulse 3 and Pulse 4 with low or no added calcium resulted in virtually no released PDGF (**Fig 5**). EGF release with Pulse 1 was strongly affected by calcium concentrations, with the highest EGF levels in supernatants when no calcium was added. The level of EGF release with Pulse 1 and no added calcium was similar to the level of EGF release with freeze/thaw treatment. Levels of EGF were low with Pulses 3–5 regardless of added calcium. The difference in released EGF *vs*. PDGF levels may suggest differences in their subcellular localization. 5-HT, which is stored in platelet dense granules, showed a pattern of release similar, but not identical, to that of PDGF. For Pulses 2, 3, and 4, which have decreasing time settings, but the same voltage setting, the levels of 5-HT decreased with decreasing time, but at each PEF condition, higher 5-HT levels were released with higher added calcium (**Table 4**).

**Table 4:** PDGF, EGF, and 5-HT in supernatants of PEF-activated PRP.

**PDGF (pg/mL)**				
	Ca^2+^_free_ 0.05 mM	Ca^2+^_free_ 0.2 mM	Ca^2+^_free_ 0.8 mM	Ca^2+^_free_ 3.0 mM
**Pulse1**	3988 ± 1714	2747 ± 1195	3081 ± 1268	2321 ± 534
**Pulse2**	2813 ± 1231	3915 ± 1699	4063 ± 1448	4221 ± 2069
**Pulse3**	655 ± 463	579 ± 330	2699 ± 2120	3270 ± 2877
**Pulse4**	631 ± 777	703 ± 830	2969 ± 2722	3515 ± 2345
**Pulse5**	3847 ± 2484	841 ± 640	6395 ± 2539	3174 ± 835
**EGF (pg/mL)**				
	Ca^2+^_free_ 0.05 mM	Ca^2+^_free_ 0.2 mM	Ca^2+^_free_ 0.8 mM	Ca^2+^_free_ 3.0 mM
**Pulse1**	1694 ± 830	1085 ± 539	866 ± 484	513 ± 249
**Pulse2**	330 ± 437	840 ± 567	821 ± 223	819 ± 363
**Pulse3**	76 ± 125	50 ± 38	116 ± 119	50 ± 63
**Pulse4**	33 ± 51	62 ± 89	280 ± 421	174 ± 239
**Pulse5**	50 ± 81	94 ± 132	117 ± 143	82 ± 136
**5-HT (ng/mL)**				
	Ca^2+^_free_ 0.05 mM	Ca^2+^_free_ 0.2 mM	Ca^2+^_free_ 0.8 mM	Ca^2+^_free_ 3.0 mM
**Pulse1**	170 ± 70	201 ± 148	300 ± 112	171 ± 85
**Pulse2**	66 ± 25	245 ± 193	369 ± 232	410 ± 282
**Pulse3**	34 ± 19	38 ± 16	169 ± 155	384 ± 349
**Pulse4**	22 ± 1	25 ± 3	127 ± 111	237 ± 199
**Pulse5**	28 ± 13	32 ± 13	279 ± 212	170 ± 144

Results shown are means ± SD, n = 3. Abbreviations: EGF, epidermal growth factor; 5-HT, serotonin; PDGF, platelet-derived growth factor; PEF, pulse electric field.

With Pulse 1 and no or low added calcium, conditions which did not result in measurable clot formation, significant amounts of PDGF, EGF, and 5-HT were released into the supernatant. Thus, release of these factors does not require clot formation. Similarly, with Pulse 1 and no or low calcium, hemoglobin release was minimal, suggesting that the release of PDGF, EGF, and 5-HT is not the result of mechanical cell breakdown.

## Discussion

The present study characterizes the effect of PEF parameters (voltage amplitude and pulse width), in combination with a range of calcium concentrations, on clot formation, hemoglobin release, growth factor release, and dense granule serotonin release. The main findings are: 1) increasing calcium concentrations with most PEF conditions leads to increased levels of PDGF and 5-HT release; 2) whether EGF levels increase or decrease with increasing calcium concentration depends on the PEF condition; 3) the pattern of PDGF and EGF levels in supernatants suggest that these molecules are localized differently within platelets; 4) significant levels of PDGF, EGF, and 5-HT can be released without inducing clot formation or hemoglobin release. Taken together, these data suggest that the combination of PEF parameters (voltage and pulse width) and calcium concentration can be used to tune the balance of growth factors, serotonin and hemoglobin released into the supernatant of PRP. Because growth factor requirements vary for different types of wounds and for wounds at different stages of healing, the unique balance of factors in supernatants of PEF-activated PRP may better meet the needs of individual clinical situations than bovine thrombin-activated PRP.

There are two main clinical protocols involving PRP: topical application of activated/clotted PRP and injection with non-activated PRP. During injections with non-activated PRP it is suggested that activation is triggered *in vivo* by collagen present at the site of the injury [21]. The experiments here demonstrated that various PEF and CaCl_2_ parameters enable growth factor release, serotonin release and minimum hemoglobin release–all without clotting. These platelet activation methods could be applied for topical uses of clotted PRP, but also for PRP injection workflows when clotting is not desirable, but growth factor and serotonin release may be beneficial.

The present study was performed using PRP that includes RBCs, sometimes called “red” PRP. Some PRP preparation devices in clinical practice produce “white” PRP [22, 23]–PRP with most RBCs removed. The results of the present study demonstrate the ability to activate “red” PRP while tuning/controlling the hemoglobin release (**Fig 2**). It should be noted that recent results suggest clinical efficacy of hemoglobin sprays in wound healing [24].

This paper quantifies for the first-time serotonin release during platelet activation with PEF. The results are surprising: serotonin release with PEF is more than 2x higher than with bovine thrombin and via freeze/thaw cycles. Of note, serotonin effects on wound healing are beneficial [25].

The electric pulse parameters and CaCl_2_ concentrations described here may not represent the optimum settings for specific workflows. In order to meet specific experimental metrics–growth factor release, clotting features, serotonin release, level of hemoglobin release, *etc*.–one may need to further optimize these parameters. Also, different PRP formulations may need additional optimization for these activation parameters via electrical stimulation, since their electrical properties may be different than the “red” PRP utilized in our study here.

While the initial motivation for pursuing electrical activation of PRP was to enable an instrument based process [26–31] that bypasses the use of thrombin and its potential side effects, cost, availability and workflow, results shown here open opportunities for tunability of PRP composition towards topical use (activated PRP, where thrombin is utilized) and injectable use (when no thrombin is utilized). It should be noted that ex vivo electric treatment of whole blood also enables platelet activation and growth factor release [32]–some clinicians that have successfully tested PRP injections for specific clinical applications, have decided to move towards whole blood injections to reduce the cost and complexity of the procedure, via bypassing the step of PRP separation from whole blood.

In conclusion, voltage, pulse width and calcium concentration can be used to control and tune the release of growth factors, serotonin and hemoglobin from PEF-activated PRP. Because growth factor requirements vary for different types of wounds and for wounds at different stages of healing, the unique balance of factors in supernatants of PEF-activated PRP may be more clinically advantageous than the current standard of bovine thrombin-activated PRP. Next steps in this research are evaluating opportunities for pilot clinical trials for wound healing using electrically activated, tunable PRP, to be completed after the ongoing Covid 19 pandemic subsides.
